# Adaptive Evolution of Energy Metabolism-Related Genes in Hypoxia-Tolerant Mammals

**DOI:** 10.3389/fgene.2017.00205

**Published:** 2017-12-07

**Authors:** Ran Tian, Daiqing Yin, Yanzhi Liu, Inge Seim, Shixia Xu, Guang Yang

**Affiliations:** ^1^Jiangsu Key Laboratory for Biodiversity and Biotechnology, College of Life Sciences, Nanjing Normal University, Nanjing, China; ^2^Comparative and Endocrine Biology Laboratory, Translational Research Institute–Institute of Health and Biomedical Innovation, School of Biomedical Sciences, Queensland University of Technology, Brisbane, QLD, Australia

**Keywords:** hypoxia-tolerance, energy metabolism, adaptive evolution, positive selection, convergent evolution

## Abstract

Animals that are able to sustain life under hypoxic conditions have long captured the imagination of biologists and medical practitioners alike. Although the associated morphological modifications have been extensively described, the mechanisms underlying the evolution of hypoxia tolerance are not well understood. To provide such insights, we investigated genes in four major energy metabolism pathways, and provide evidence of distinct evolutionary paths to mammalian hypoxia-tolerance. Positive selection of genes in the oxidative phosphorylation pathway mainly occurred in terrestrial hypoxia-tolerant species; possible adaptations to chronically hypoxic environments. The strongest candidate for positive selection along cetacean lineages was the citrate cycle signaling pathway, suggestive of enhanced aerobic metabolism during and after a dive. Six genes with cetacean-specific amino acid changes are rate-limiting enzymes involved in the gluconeogenesis pathway, which would be expected to enhance the lactate removal after diving. Intriguingly, 38 parallel amino acid substitutions in 29 genes were observed between hypoxia-tolerant mammals. Of these, 76.3% were radical amino acid changes, suggesting that convergent molecular evolution drives the adaptation to hypoxic stress and similar phenotypic changes. This study provides further insights into life under low oxygen conditions and the evolutionary trajectories of hypoxia-tolerant species.

## Introduction

Ever since Charles Darwin published his book ‘On the Origin of Species by Means of Natural Selection’ nearly 160 years ago (Darwin, 1859), adaptive evolution has remained a critical research question. Of particular interest are insights from hypoxia-tolerant animals – species adapted to oxygen (O_2_) poor aquatic or terrestrial environments (Nathaniel et al., 2015). Oxygen is vital to animals and hypoxia is associated with cell death and organ failure, as observed in stroke and ischemia reperfusion injury in humans (Buck and Pamenter, 2006).

Marine mammals include approximately 120 species, spanning three distinct mammalian orders which independently transitioned to an aquatic environment: Cetacea (whales, dolphins, and porpoises), Pinnipedia (walruses, sea lions, and other pinnipeds), and Sirenia (manatees and dugongs) (Hoelzel, 2002; Berta et al., 2006). Hypoxia-tolerance is one of the major features of marine mammals. Reduced metabolism, hypometabolism, in marine mammals is associated with an increased reliance on anaerobic (‘without oxygen’; nitrate) metabolism; the advantage of which is that energy in the form of adenosine triphosphate (ATP) can be produced in the absence of oxygen (Costa, 2007). Marine animals also display an enhanced capacity of glial cells to support anaerobic breakdown of glucose, glycolysis, coupled with an elevated number of astrocytes which store glycogen and provide lactate as an energy source (Ramirez et al., 2007). Higher concentrations or activity of key glycolytic enzymes that enhance the ability to process lactic acid, such as lactate dehydrogenase (LDH), may be associated with greater tolerance to hypoxia (Costa, 2007).

Mammals that live at high altitude are also exposed to hypoxic environments. To adapt to high-altitude hypoxia, for instance, Tibetan humans have a greatly reduced abundance of mitochondrial DNA in muscle (the number of mitochondrial genomes present), indicative of a reduced metabolic capacity (Storz et al., 2010). Moreover, alteration in enzyme kinetics, e.g., higher substrate affinity of cytochrome c oxidase enzyme (COX), may enhance hypoxia-tolerance by preventing oxidative damage in high-altitude species (Storz et al., 2010). In addition, exclusively subterranean species, such as the naked mole rat (*Heterocephalus glaber*), live in oxygen-poor burrows (Bennett and Faulkes, 2000). The naked mole rat has developed adaptive metabolic functions, such as a reduced metabolic rate and retarded somatic development (neoteny) (Fang et al., 2014; Park et al., 2017).

Molecular evolution of genes associated with energy metabolism has attracted much interest in recent decades. For example, evidence of positive selection was found in genes in the mitochondrial oxidative phosphorylation (OXPHOS) pathway (the final step in the production of ATP from the oxidation of nutrients) in two snakes and bats-burrowing and flying animals (Castoe et al., 2008; Shen et al., 2010; Castoe et al., 2013); suggesting that evolution of energy metabolism genes are critical for acclimation to novel environments. Here, to provide additional evidence on the evolution of energy metabolism genes in hypoxia-tolerant mammals, we investigated coding sequences of 194 energy metabolism-related genes across diverse mammalian taxa, aquatic and terrestrial hypoxia-tolerant species.

## Materials and Methods

### Sequences Retrieval and Alignments

**FIGURE 1:**
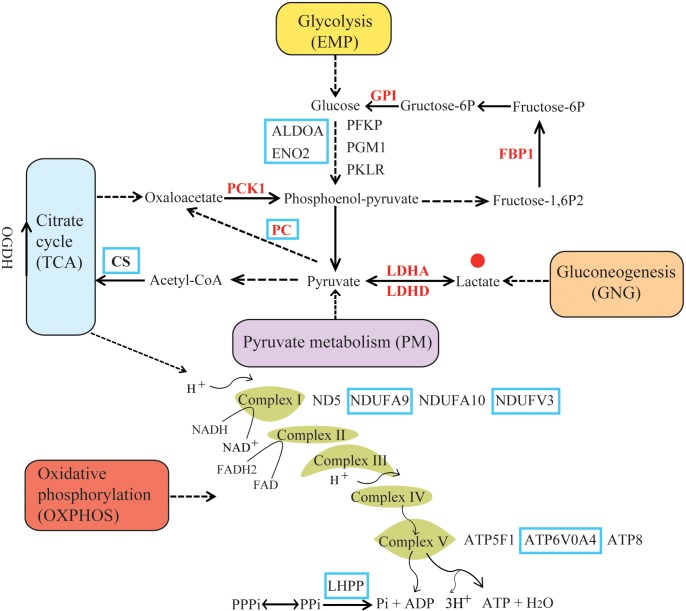
Summary diagram of energy metabolism pathway. Pathway categories were obtained from KEGG. Positively selected genes (PSGs) identified in hypoxia-tolerant mammals are shown in red. The solid lines indicate direct relationships between enzymes and metabolites. The dashed lines indicate that more than one step is involved in a process. Glycolysis (EMP) metabolizes glucose to pyruvate, which provides the cell with ATP under anaerobic conditions (Fromm and Hargrove, 2012). In contrast, gluconeogenesis (GNG) drives the synthesis of D-glucose from non-carbohydrate sources (e.g., lactate), and some glycolytic enzymes are part of the gluconeogenesis pathway. In the pyruvate metabolism (PM) pathway, pyruvate is processed anaerobically (fermented) to lactate (lactic acid fermentation) or ethanol (alcoholic fermentation), and can be aerobically oxidized to CO_2_. Of note, lactate and pyruvate is either the product of EMP or the major precursor for GNG; moreover, the former can be converted back to pyruvate when conditions return to normal, or excreted in the urine (Pelley, 2007). The citrate cycle (TCA) contains genes responsible for the conversion of pyruvate to acetyl CoA under aerobic conditions. Oxidative phosphorylation (OXPHOS) is composed of multi-subunit complexes, encoded by both nuclear and mitochondrial genes, which catalyzes the synthesis of ATP (Berg et al., 2002). In the absence of sufficient oxygen, electron transport ceases and the energy demands of the cell are not maintained (Giordano, 2005).

A list of human energy metabolism-related genes was retrieved from the KEGG (Kyoto Encyclopedia of Genes and Genomes) database (Kanehisa and Goto, 2000). The following pathways were retrieved: Glycolysis/Gluconeogenesis (EMP/GNG): ko00010; Citrate cycle: ko00020 (TCA); Pyruvate metabolism (PM): ko00620; and Oxidative phosphorylation (OXPHOS): ko00190 (**Figure 1**). The resulting gene list was used to query Ensembl BioMart and retrieve non-human Ensembl gene identificators (Kinsella et al., 2011). The gene IDs were used to search annotated coding sequences (CDS) of mammals in the NCBI^1^ and Ensembl databases^2^ (Supplementary Table S1). Additionally, BLASTn and tBLASTn searches (Johnson et al., 2008) were performed to identify one-to-one orthologs in the genomes of 12 hypoxia-tolerant species. This included 9 marine mammals, cetaceans: bottlenose dolphin (*Tursiops truncatus*), baiji (*Lipotes vexillifer*), killer whale (*Orcinus orca*), sperm whale (*Physeter macrocephalus*), bowhead whale (*Balaena mysticetus*), minke whale (*Balaenoptera acutorostrata*); pinnipeds: Weddell seal (*Leptonychotes weddellii*), Pacific walrus (*Odobenus rosmarus divergens*), sirenians: West Indian manatee (*Trichechus manatus latirostris*); 2 highland mammals: Tibetan yak (*Bos mutus*), Tibetan antelope (*Pantholops hodgsonii*); and 1 subterranean mammals: naked mole rat (*Heterocephalus glaber*) (Supplementary Figure S1). For genes with multiple splice variants, the longest transcript was retained. The resulting dataset, derived from 54 mammalian genomes, included 181 nuclear-encoded energy metabolism-related genes (Supplementary Table S1). In addition, nucleotide sequences of 13 mitochondrial protein-coding genes from 101 species were downloaded from NCBI and the MitoZoa database^3^ (Supplementary Figure S2). A total of 194 energy metabolism-related (nuclear and mitochondrial) genes were obtained. The EMP/GNG, PM, TCA, and OXPHOS metabolic pathways were composed of 49, 34, 29, and 111 genes, respectively (Supplementary Figure S3). Genes shared by different pathways are depicted in Supplementary Figure S3.

Coding (amino acid) sequence for each gene was aligned using MUSCLE v3.2 (Edgar, 2004) with default settings, manually corrected, and used to align nucleotide sequences in MEGA 6 (Tamura et al., 2013). Because the quality of sequence alignments dramatically affects the estimation of evolutionary parameters, we deleted all gaps and undetermined bases ‘N’ in alignments to reduce the rate of false-positive prediction, and only high-quality sequences were used in downstream analyses. The species used for each gene, and associated accession numbers, are presented in Supplementary Table S1.

### Molecular Evolution Analyses

The impact of natural selection on energy metabolism related genes was determined by estimating the ratio of non-synonymous (*d*_N_) and synonymous (*d*_S_) mutations (ω = *d*_N_/*d*_S_) implemented in CodeML (implemented in PAML v4.8) (Yang, 2007). Briefly, ω > 1, ω = 1, and ω < 1 indicate positive selection, neutral, and negative selection, respectively. To determine whether codon positions in hypoxia-tolerant branches were under positive selection, we employed the PAML branch-site model, which assesses selective pressure (ω) at sites in foreground lineages of interest, specified *a priori*, and remaining background lineages (Zhang et al., 2005). In separate tests, branches leading to cetaceans, pinnipeds, sirenians, highland (high-altitude) species, or subterranean (burrowing) mammals were specified as foreground branches, with the remaining of the tree as the background branch (Supplementary Figures S1, S2). Additionally, we also analyzed the combined branches, all hypoxia-tolerant mammals, independently. Compared with the corresponding null hypothesis of neutral evolution, foreground branches in the modified model A (test 2) that have a class of sites with the ratio ω_2_ > 1 are candidates for positive selection. Likelihood ratio tests (LRTs) with a chi-squared test (df = 2) were conducted on nested models. Such sites under positive selection were detected by the Bayes empirical Bayes (BEB) method, with posterior probabilities of ≥0.80 (Yang et al., 2005). Resulting *P*-values were corrected by employing a false discovery rate (FDR) cutoff of 0.05 (Storey and Tibshirani, 2003). A well-accepted species tree among mammals (Supplementary Figures S1, S2) was used as the input tree in all analyses (Murphy et al., 2001).

### Parallel/Convergent Site Detection

In order to identify parallel/convergent amino acid changes in hypoxia-tolerant mammals, the sequences of the ancestral nodes were reconstructed with the help of the CodeML program implemented in PAML (Yang, 2007). Convergent/parallel amino acid replacements, from the last common ancestor-state to present-species along each hypoxia-tolerant branch, were detected by an in-house Python script (Supplementary Data Sheet 1). Amino acid residue changes were classified into convergent or parallel changes. Convergent changes: any amino acid at the same position changes to the same amino acid in two or more distinct lineages (for example, Ala10Glu in cetaceans; His10Glu in pinnipeds). Parallel changes: an identical amino acid at the same position changes to the same amino acid in two or more distinct lineages (for example, Ala10Glu in cetaceans; Ala10Glu in pinnipeds). To test whether observed parallel/convergent substitutions in focal hypoxia-tolerant branches were fixed by random chance or by natural selection, the statistical significance of amino acid changes was tested by the method developed by Zhang and Kumar (). To carry out in-depth investigation of convergence between hypoxia-tolerant species, we measured the fit (site-wise log-likelihood support; SSLS) (Parker et al., 2013) of each amino acid along the orthologous CDS alignment to the generally accepted species tree (H_0_) and a convergent tree (H_A_) that constrained hypoxia-tolerant species into monophyletic clades. The difference in SSLS for a single site under different tree topologies was calculated as follows: ΔSSLS = SSLS (H_A_) - SSLS (H_0_) (Supplementary Figure S4), where a negative ΔSSLS implies support for convergence. SSLS for each gene alignment was calculated by RAxML v8.0.26 (Stamatakis, 2014).

### Liver Transcriptome Analyses

The expression of the 194 energy metabolism-related genes were examined in *de novo* assembled transcriptomes (Seim et al., 2014). We contrasted cetacean (bowhead whale and minke whale) and terrestrial (naked mole rat, domestic yak, Brandt’s bat, Chinese tree shrew, rhesus monkey, rat, and mouse) liver transcriptomes. Briefly, library size-normalized read counts were subjected to the *voom* function (variance modeling at the observation-level) function in the R package ‘limma’ v3.18.13 (Law et al., 2014) with *trend = TRUE* for the eBayes function and correction for multiple testing (Benjamini–Hochberg false discovery *P* ≤ 0.05). Following limma analysis, genes with a log_2_ 1.2-fold-change differences between cetaceans (bowhead whale, and minke whale) and other mammals were considered differentially expressed. RNA-seq gives very accurate measurements of relative expression across a broad dynamic range (Wang et al., 2008). However, in cross-species analyses (here, two cetaceans versus seven terrestrial mammals), one would expect more heterogeneity than when comparing within a species (where typically a log_2_ 2.0-fold-change cutoff is employed). Thus, a gene could be modestly upregulated, but still be biologically relevant.

## Results

### Selective Regimes on Energy Metabolism Related Genes Imposed by Hypoxia-Tolerant Species

**FIGURE 2:**
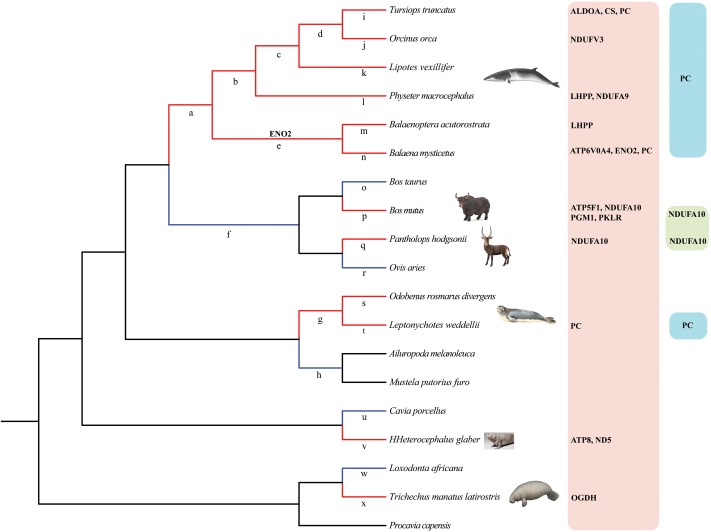
Phylogenetic tree representation of energy metabolism genes under positive selection in hypoxia-tolerant mammals. Red and blue color branches in the tree denote hypoxia-tolerant mammals and their sister taxa, respectively. Branches a-x in the tree were used in branch-site tests. PSGs (*PC*, *NDUFA10*) shared by two or more hypoxia-tolerant species were also listed in the rightmost part of the **Figure 2**.

To estimate the selective pressure acting on genes in energy metabolism pathways in mammals, we ran separate branch-site tests on the ancestral and terminal branches leading to cetaceans (bottlenose dolphin, baiji, killer whale, sperm whale, minke whale, and bowhead whale), pinnipeds (Weddell seal and Pacific walrus), sirenian (West Indian manatee), highland species (Tibetan yak, and Tibetan antelope), and a subterranean species (naked mole rat) (**Figure 2** and Supplementary Table S2). In cetaceans, eight genes showed evidence of positive selection (positively selected genes; PSGs) (test 2, Supplementary Table S2) along the lineages leading to bottlenose dolphin (**Figure 2**: branch i, *ALDOA*, *CS*, and *PC*), killer whale (**Figure 2**: branch j, *NDUFV3*), sperm whale (**Figure 2**: branch l, *LHPP*, and *NDUFA9*), minke whale (**Figure 2**: branch m, *LHPP*), bowhead whale (**Figure 2**: branch n, *ATP6V0A4*, *ENO2*, and *PC*), and the last common ancestor of baleen whales (mysticetes; **Figure 2**: branch e, *ENO2*) (**Table 1**) after correcting for multiple testing. For the remaining aquatic mammals, the LRT test showed evidence for positive selection on the genes *PC* and *OGDH*, respectively, in the lineages leading to Weddell seal (**Figure 2**: branch t) and West Indian manatee (**Figure 2**: branch x). When considering highland species, one gene (*NDUFA10*) was under positive selection in the Tibetan antelope (**Figure 2**: branch q), whereas in Tibetan yak four genes (*PGM1*, *PKLR*, *ATP5F1*, and *NDUFA10*) were under positive selection (**Figure 2**: branch o). When we set the subterranean (naked mole rat) as the foreground branch (**Figure 2**: branch v), positive selection was detected in two mitochondrial genes: *ATP8* and *ND5*. Notably, the PSGs identified in hypoxia-tolerant species were not found in their sister taxa, although some genes in the sister taxa were also positively selected (Supplementary Table S2). These finding suggests that the PSGs identified are robust candidate hypoxia adaptation genes.

**Table 1:** Positively selected genes (PSGs) in hypoxia-tolerant mammals after multiple-testing correction (FDR).

Order	Species	Habitat	Total PSGs numbers	PSGs	Numbers/Proportions (%) of PSGs in pathway
Cetacea	cetacean	Marine	8	*ALDOA, ENO2*	EMP/GNG 2 (4.1)
				*CS, PC*	TCA 2 (6.9)
				*PC*	PM 1 (2.9)
				*ATP6V0A4, LHPP, NDUFA9, NDUFV3*	OXPHOS 4 (3.6)
Pinnipedia	Weddell seal	Marine	1	*PC*	TCA 1 (3.4)
				*PC*	PM 1 (2.9)
Sirenia	West Indian manatee	Marine	1	*OGDH*	TCA 1 (3.4)
Artiodactyla	Tibetan yak	Highland	4	*PGM1, PKLR*	EMP/GNG 2 (4.1)
				*PKLR*	PM 1 (2.9)
				*ATP5F1, NDUFA10*	OXPHOS 2 (1.8)
Artiodactyla	Tibetan antelope	Highland	1	*NDUFA10*	OXPHOS 1 (0.9)
Rodentia	naked mole rat	Subterranean	2	*ATP8, ND5*	OXPHOS 2 (1.8)

*EMP/GNG, TCA, PM, and OXPHOS represent pathway of Glycolysis/Gluconeogenesis, Citrate cycle pathway, Pyruvate metabolism, and Oxidative phosphorylation in KEGG database, respectively*.

### Energy Metabolism Pathway Clustering of PSGs in Hypoxia-Tolerant Species

**FIGURE 3:**
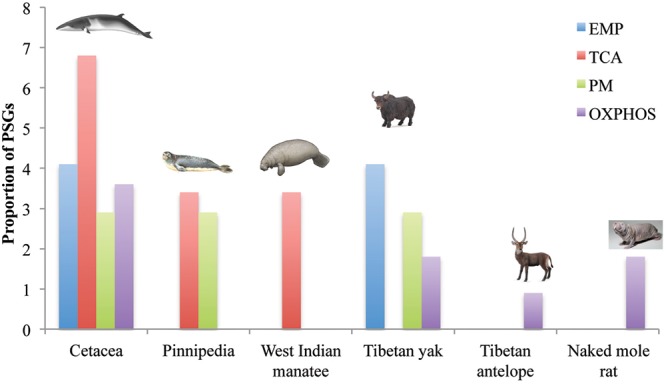
Positively selected genes of four energy metabolism pathways in hypoxia-tolerant mammals. EMP/GNG, TCA, PM, and OXPHOS denote the KEGG Pathway categories Glycolysis/Gluconeogenesis, Citrate cycle (TCA cycle), Pyruvate metabolism, and Oxidative phosphorylation, respectively.

To assess the potential function of PSGs in hypoxia-tolerant mammals, we categorized them at the pathway level. In cetaceans, PSGs were found in all four energy metabolism pathways of interest: 6.9% (2/29 genes) in TCA, 4.1% (2/49 genes) in EMP/GNG, 3.6% (4/111 genes) in OXPHOS, and 2.9% (1/34 genes) in PM (**Figure 3** and **Table 1**). Notably, PSGs were enriched in TCA signaling pathway in the cetacean lineages, with approximately twice as many PSGs as the PM pathway (**Figure 3** and **Table 1**). Similarly, PSGs were also enriched in TCA signaling pathway in the pinniped and sirenian branches (**Figure 3**). In contrast, PSGs in the Tibetan antelope (0.9%, 1/111 genes) and naked mole rat (1.8%, 2/111) were exclusively found in the OXPHOS pathway. Tibetan yak showed positive selection in EMP/GNG (4.1%, 2/49 genes), PM (2.9%, 1/34 genes) pathways, and OXPHOS (1.8%, 2/111 genes) pathways.

### Evidence for Species-Specific Adaptations in Cetacean Energy Metabolism Related Genes

To gather additional evidence on the adaptive evolution of energy metabolism related genes in cetaceans, we reconstructed ancestral sequences and mapped species-specific amino acid changes along cetacean lineages. Compared with other mammals, a total of 55 unique amino acid substitutions in 44 genes were observed in all cetaceans; three of which (*ATP6V0A4*, *ENO2*, and *PC*) were inferred to have evolved under positive selection (Supplementary Table S3). Given that differential gene expression could conceivably also contribute to hypoxia-tolerance (Böhne et al., 2013; Fang et al., 2014), we contrasted liver (a key metabolic organ) transcriptomes from two cetaceans (bowhead whale, and minke whale) and seven terrestrial mammals, a liberal cutoff (log_2_ fold-change ≥ 1.2) revealed no significant differential expression of the 194 energy metabolism genes interrogated in this study (Supplementary Figure S5 and Supplementary Table S4).

### Identification of Parallel/Convergent Substitutions in Hypoxia-Tolerant Mammals

**FIGURE 4:**
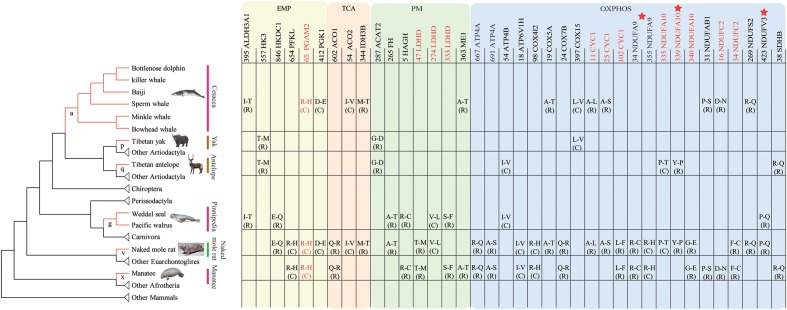
Parallel/convergent amino acid substitutions specifically occurred along hypoxia-tolerant species. Red sites represent parallel non-synonymous amino acid substitutions occurred along more than 3 hypoxia-tolerant lineages; Stars represent PSGs. Branch a represented the last common ancestor (LCA) of cetaceans, g of the LCA of pinnipeds, x of West Indian manatee, p of the Tibetan yak, q of the Tibetan antelope, and v of the naked mole rat, respectively.

To identify convergent evolution of energy metabolism related genes in hypoxia-tolerant mammals from different habitats, we reconstructed ancestral gene sequences for the internal nodes of the species tree and identified shared amino acid substitutions along lineages leading to hypoxia-tolerant species (**Figure 4**). In total, 7 parallel amino acid residue substitutions in 7 genes were identified in the marine group (branch a, g, x), as well as 2 parallel substitutions in 2 genes in high-altitude species (branch p, q). We identified 26, 3 and 2 parallel substitutions in 23, 3, and 1 genes in marine + subterranean mammals (branch v), marine + highland mammals, and highland + subterranean mammals, respectively (**Table 2**). Taken together, 38 parallel non-synonymous amino acid substitutions in 29 genes occurred along hypoxia-tolerant species. The number of substitutions was not random (*P* ≤ 0.05; data not shown). Three of these genes (*NDUFA9*, *NDUFA10*, and *NDUFV3*) were inferred to have evolved under positive selection in the hypoxia-tolerant lineages. Remarkably, parallel non-synonymous amino acid substitutions in 5 genes (*CYC1*, *LDHD*, *NDUFA10*, *NDUFC2*, and *PGAM2*) occurred along more than two hypoxia-tolerant species lineages (**Table 2**). Moreover, all 38 substitutions also ranked highly in the convergence signal distribution, and 92.1% (35/38) loci in the top 5% supporting convergent tree by ΔSSLS test (**Table 2**). Additionally, 76.3% (29/38) of substitutions were radical amino acid changes.

**Table 2:** Thirty-eight parallel amino acid substitutions in 29 genes in hypoxia-tolerant species.

Branches	Gene name	Pathway	Site	ΔSSLS	AA change	Radical/Conservative changes	Functional sites (identity with sites of human)
a vs. p	*COX15*	OXPHOS	397	**Y**	L-V	C
a vs. g	*ALDH3A1*	EMP/GNG	395	**Y**	I-T	R
a vs. v	*ACO2*	TCA	54	**Y**	I-V	C	Modified residue: 50, N6-acetyllysine; alternate
	*COX5A*	OXPHOS	19	**Y**	A-T	R
	***CYC1***	OXPHOS	11	**Y**	A-L	R
			25	**Y**	A-S	R
	*IDH3B*	TCA	344	**Y**	M-T	R
	*NDUFS2*	OXPHOS	269	**Y**	R-Q	R
	***PGAM2***	EMP/GNG	65	**Y**	R-H	C
	*PGK1*	EMP/GNG	412	**Y**	D-E	C
a vs. x	*ME1*	PM	363	**Y**	A-T	R
	*NDUFAB1*	OXPHOS	31	**Y**	P-S	R	Transit peptide 1-68
	***NDUFC2***	OXPHOS	16	**Y**	D-N	R
	***PGAM2***	EMP/GNG	65	**Y**	R-H	C	Binding site: 62, Interaction with carboxyl group of phosphoglycerates
p vs. q	*ACAT2*	PM	287	**Y**	G-D	R
	*HK3*	EMP/GNG	557	**Y**	T-M	R	Domain: Hexokinase 2: 477–912; region: 489–923: Catalytic; region: 531–661: Hexokinase small subdomain 2
q vs. v	***NDUFA10***	OXPHOS	335	Y	P-T	C
			339	Y	Y-P	R
g vs. q	*ATP4B*	OXPHOS	54	**Y**	I-V	C
g vs. v	*FH*	TCA, PM	265	**Y**	A-T	R
	*HKDC1*	EMP/GNG	846	**Y**	E-Q	R	Domain: Hexokinase 2: 464–905; region: Hexokinase large subdomain 2: 655–894
	***LDHD***	PM	274	**Y**	V-L	C
	*NDUFV3*	OXPHOS	423	**Y**	P-Q	R
g vs. x	*HAGH*	PM	5	**Y**	R-C	R
	***LDHD***	PM	333	**Y**	S-F	R	Modified residue: 358, N6-acetyllysine; alternate
x vs. q	*SDHB*	TCA, OXPHOS	38	Y	R-Q	R
x vs. v	*ACO1*	TCA	602	**Y**	Q-R	R
	*ATP4A*	OXPHOS	667	**Y**	R-Q	R
			691	**Y**	A-S	R
	*ATP6V1H*	OXPHOS	18	**Y**	I-V	C
	*COX4I2*	OXPHOS	98	**Y**	R-H	C
	*COX7B*	OXPHOS	24	**Y**	Q-R	R
	***CYC1***	OXPHOS	102	**Y**	L-F	R
	***LDHD***	PM	47	**Y**	T-M	R	Domain: FAD-binding PCMH-type: 62–265
	*NDUFA9*	OXPHOS	34	**Y**	R-C	R	Transit peptide: Mitochondrion: 1–35; chain: 36–377 NADH dehydrogenase [ubiquinone] 1 alpha subcomplex subunit 9, mitochondrial
			355	**Y**	R-H	C
	***NDUFA10***	OXPHOS	340	**Y**	G-E	R	Chain: 36–355: NADH dehydrogenase [ubiquinone] 1 alpha subcomplex subunit 10, mitochondrial)
	***NDUFC2***	OXPHOS	34	**Y**	F-C	R
	*PFKL*	EMP/GNG	654	**Y**	M-L	C
	***PGAM2***	EMP/GNG	65	**Y**	R-H	C

*EMP/GNG, TCA, PM, and OXPHOS denote the KEGG Pathway categories Glycolysis/Gluconeogenesis, Citrate cycle, Pyruvate metabolism, and oxidative phosphorylation, respectively. Branch a: ancestor of cetacean, branch p: Tibetan yak, branch q: Tibetan antelope, branch g: ancestor of pinnipedia, branch v: naked mole rat, branch x: manatee were consistent with branches in **Figure 4**. Functional sites were identified using UniProt (http://www.uniprot.org). Bold genes occurred along more than two hypoxia-tolerant species. Underlined genes were under positive selection. In the table column ΔSSLS, Y denotes genes with a positive ΔSSLS score (bolded if ranked in the top 5% of genes tested)*.

## Discussion

### Genetic Discrepancy between Hypoxia-Tolerant Species

We found evidence of positive selection acting on energy metabolism related genes in diverse hypoxia-tolerant species. PSGs related to the OXPHOS pathway mainly occurred along hypoxia-tolerant lineages with a terrestrial lifestyle (such as Tibetan yak, Tibetan antelope, and naked mole rat), whereas PSGs associated with the TCA pathway appeared to have a stronger signal of positive selection in marine mammals. Species-specific PSGs were examined in the hypoxia-tolerant mammals, providing useful pointers to the evolution of unique traits. For example, *ALDOA*, *ENO2*, *CS*, *ATP6V0A4*, *LHPP*, *NDUFA9*, and *NDUFV3* PSGs were unique to cetaceans.

Another PSG (*PC*) found in two cetaceans and the Weddell seal encodes pyruvate carboxylase, an enzyme which converts pyruvate to oxalacetic acid - the first control step of gluconeogenesis (Söling and Willms, 2013). Adaptive evolution of this gene suggests that a hypoxia adaptation of marine mammals involves the Cori cycle and improved lactic acid metabolism during recovery from dives (Cori, 1981; Castellini, 2012). Similarly, *PKLR*, which encodes pyruvate kinase, a rate-limiting enzyme in glycolysis, was unique to Tibetan yak. This finding is consistent with a previous study on this species (Ge et al., 2013), suggesting that glycolysis is essential for survival in high-altitude hypoxic environments. Two mitochondrial genes (*ND5* and *ATP8*) in the OXPHOS pathway were exclusively identified to be under positive selection in the naked mole rat, suggesting that mitochondria play a role in subterranean hypoxia-tolerance. Various studies have investigated hypoxia-tolerance in the naked mole rat (Fang et al., 2014; Park et al., 2017), but its mitochondrial energetics remains largely unexplored, which are likely critical to its hypoxia-tolerance and extraordinary lifespan (Stoll et al., 2016).

The above observations are indicative of species- or lineage-specific adaptations to hypoxia. There are a several, non-mutually exclusive explanations for this deduction. It is well established that physiological features supporting a hypoxic lifestyle have evolved independently. Marine mammals routinely experience acute hypoxia, which causes them to depend on a finite supply of intrinsic oxygen stores (Ramirez et al., 2007). A dive is manifested by progressive asphyxia – concomitant with increased hypoxia, hypercapnia, and acidosis (Elsner and Gooden, 1983) – yet marine mammals exhibit an extraordinary ability to circumvent the deleterious effects of hypoxia. These include apnea (cease of breath), bradycardia, and subsequent decreased cardiac output, as well as peripheral vasoconstriction (selective shutdown of blood flow in non-essential, peripheral tissues) (Panneton, 2013). In contrast to marine mammals, terrestrial animals that live at high-altitudes or in burrows cope with a different form of hypoxic stress, chronic hypoxia due to a lower partial oxygen pressure (pO_2_), by improving O_2_ affinity and increasing ventilation and cardiac output (Ramirez et al., 2007). For example, the genomes of the Tibetan antelope and Tibetan yak show independent signals of adaptive evolution and gene-family expansion. Genes associated with oxygen transmission and mitochondrial membranes expanded in Tibetan antelope (Ge et al., 2013), whereas genes related to sensory perception and olfactory receptor activity expanded in Tibetan yak (Qiu et al., 2012), respectively. A series of genome scans of high-altitude mammals have identified genes under positive selection: *EGLN1* and *EPAS1* in Tibetan humans (Simonson et al., 2010; Yi et al., 2010); and *ADAM17*, *ARG2*, and *MMP3* in Tibetan yak (Qiu et al., 2012). Moreover, candidate high-altitude gene loci in snub-nosed monkey populations are manifested by habitat-specific molecular evolution (Yu et al., 2016). Taken together, ecological and/or biogeographic differences, as well as distinct physiological traits, may have imposed divergent selective pressures on the evolution of hypoxia-tolerance.

### Further Insights into Lineage-Specific Evolution of Hypoxia-Tolerance from Cetaceans

Cetaceans, one of three extant marine mammal orders descended from terrestrial ancestors that recolonized the sea, offer an ideal case study. During the habitat transition of cetacean ancestors, dramatic changes in anatomy and physiology occurred, e.g., streamlined body, paddle-shaped fore-flippers, degenerated hindlimbs, and lack of hair (Berta et al., 2006; Muizon, 2009). Cetaceans are also further challenged by acute episodes of hypoxia during diving. Cetacean adaptations to hypoxia are manifested as increased oxygen storage in blood and muscle, a reduced metabolic rate, and selective vasoconstriction (Costa, 2007; Ramirez et al., 2007). Molecular mechanisms underlying these innovations have only begun to be explored (Mirceta et al., 2013; McGowen et al., 2014; Tian et al., 2016).

In this study, we also identified genes under positive selection in cetaceans and investigated their functional enrichment, at the energy metabolism pathway level. We found a signature of positive selection across cetaceans in a total of eight genes, and in all four energy metabolism pathways (EMP/GNG: 2; TCA: 2; PM: 1; and OXPHOS: 4). Interestingly, the highest proportion of PSGs was in the TCA cycle. The TCA cycle is a central part of aerobic metabolism and a chief source of electrons for ATP generation from the chemical breakdown of carbohydrates, fats, and proteins (Weitzman, 1987). Of note, this includes the citrate synthase (*CS*) gene, which encodes a key rate-limiting enzyme in energy production via the TCA cycle. We speculate that these changes mediate an enhanced capability for aerobic metabolism by cetaceans during dives (Castellini, 2012). Of particular interest, six genes (*LDHA*, *LDHD*, *PC*, *PCK1*, *FBP1*, and *GPI*) with cetacean-specific amino acid changes are rate-limiting enzymes in the gluconeogenesis pathway (GNG) (Cori, 1981) (**Figure 1** and Supplementary Figure S6). Changes in these genes may result in improved conversion of lactate to glucose or glycogen; clearing lactate from the blood and restoring circulating levels of glucose. Two genes (*ALDOA* and *ENO2*) associated with anaerobic respiration adaptively evolved in cetaceans, further supporting the role of glycolysis (EMP/GNG pathway) as a critical energy supply during prolonged, deep dives (Butler and Jones, 1997).

It is appreciated, however, that stratifying hypoxia-resistance and other unique adaptations at the genetic level is not straightforward. Cetaceans have large, energy-demanding brains and previous genome-wide studies of mammals revealed that genes associated with energy metabolism are targets of natural selection in cetaceans (McGowen et al., 2012; Shen et al., 2012; Sun et al., 2013). It then follows that selection acting on energy metabolism-related genes may be in response to the evolution of hypoxia adaptation, large brains, as well as aquatic locomotion (reflecting the historic shift from running to swimming). Similarly, flying insects and mammals (bats) also require significant energy for locomotion. Indeed, studies on bats (Shen et al., 2010, 2012) revealed a critical role for genes in the OXPHOS pathway in flight. Previous molecular studies on primates also showed evidence of adaptive evolution in energy metabolism-related genes, suggesting that these genes played a major role in overcoming elevated energy demands during the expansion of the neocortex (Grossman et al., 2001). Taken together, positive selection of energy metabolism-related genes in non-hypoxia-tolerant species is expected, and not mutually exclusive to a role in hypoxia-tolerance, given that such genes had multiple advantageous phenotypic effects (pleiotropy) during mammalian evolution.

### Convergent Evolution of Energy Metabolism-Related Genes in Hypoxia-Tolerant Species

Conceptually, hypoxia-tolerance provides a classical example of convergent evolution. Even though there are clearly environmental differences, inherent similarities may drive recurrent evolution at the sequence level, manifested as convergent or parallel amino acid changes. Recent work, relying on whole-genome sequences of marine mammals (Foote et al., 2015), revealed that convergent amino acid substitutions in genes associated with phenotypic adaptations can be identified. It is appreciated that hypoxia-tolerance is a very complex phenomenon that involves a suite of genes. Although the morphologies and physiological characteristics of hypoxia-tolerance in marine and terrestrial mammals differ greatly, we reveal sequence convergence in energy metabolism-related genes. These genes could reflect core hypoxia-tolerance associated functions. Interestingly, one PSG (*PFKP*) was detected in the ‘combined’ hypoxia-tolerant mammal branches (Supplementary Table S1). Five genes (*NDUFA9*, *NDUFA10*, *NDUFAB1*, *NDUFC2*, and *NDUFV3*) encodes the nuclear-encoded subunits of complex I in the OXPHOS pathway (**Figure 1**). Parallel/convergent evolution of these gene may reflect the importance of oxidative phosphorylation for survival in hypoxic environments. Hypoxia also induces oxidative stress, manifested by lipid peroxidation, protein oxidation, and DNA damage (Ramirez et al., 2007). *ALDH3A1* (encodes cytosolic aldehyde dehydrogenase 3A1; **Figure 1**) protects against oxidative stress in various ways, including enhanced DNA and cell repair (Estey et al., 2007). Another gene, *PFKP* (phosphofructokinase platelet type; **Figure 1**), catalyzes the glycolytic pathway by converting fructose-6-phosphate to fructose-1, 6-bisphosphate (Webb et al., 2015). Mutations inhibiting phosphofructokinase activity cause glycogen storage disease (Webb et al., 2015). Taken together, we reveal several convergent genes in hypoxia-tolerant species.

## Conclusion

In summary, in this study we investigated genes in four major, interconnected energy metabolism pathways in hypoxia-tolerant marine and terrestrial mammals. We reveal distinct selection of a 194-gene set in hypoxia-tolerant species. Moreover, we report cetacean-specific amino acid changes in the rate-limiting enzymes of the gluconeogenesis (GNG) pathway that likely enhance lactate removal following hypoxic dives. Surprisingly, we found evidence of convergent evolution of a subset of genes in hypoxia-tolerant mammals, a phenomenon which could reflect phenotypic convergence. Our work reveals a number of candidate hypoxia-tolerance genes which can be further explored by experimental and computational analyses.

## Ethics Statement

All methods used to collect observational data were non-invasive and in compliance with the regulations and guidelines of the Nanjing Normal University Institutional Animal Care and Use Committee.

## Author Contributions

GY and SX conceived and designed the study. RT, DY, and YL collected the data. RT and IS conducted the bioinformatics analyses. RT wrote the manuscript, and GY, SX, and IS revised the manuscript. All authors read and approved the final manuscript.

## Conflict of Interest Statement

The authors declare that the research was conducted in the absence of any commercial or financial relationships that could be construed as a potential conflict of interest.

## Footnotes

1 http://www.ncbi.nlm.nih.gov

2 http://www.ensembl.org

3 http://srv00.recas.ba.infn.it/mitozoa/
