# Supplementary material for: Adaptive Evolution of Energy Metabolism-Related Genes in Hypoxia-Tolerant Mammals
Source: Front Genet. 2017 Dec 7;8:205. doi: 10.3389/fgene.2017.00205 (PMC5725996; doi:10.3389/fgene.2017.00205)
Supplement: Supplementary file 5 [file Data_Sheet_1.DOCX]

**Python script for identifying convergent/parallel amino acid replacements.**

#!/usr/bin/env python

# -*- coding: utf-8 -*-

from Bio import Align

from Bio import SeqIO, AlignIO

def parallel_convergent_batch(infile='*.fas',outfile='*.csv',sep='\t',a1='',b1='',c2='',d2=''):

#0: Divergent 1: Parrallel 2: Convergent -1: Gaps -2: Others

#a1: ancestral node number of sequences 1

#b1: node number of sequences 1

#c2: ancestral node number of sequences 2

#d2: node number of sequences 2

s=SeqIO.to_dict(SeqIO.parse(infile,'fasta'))

s1=s[a1].seq

p1=s1.translate(table=1)

s2=s[b1].seq

p2=s2.translate(table=1)

s3=s[c2].seq

p3=s3.translate(table=1)

s4=s[d2].seq

p4=s4.translate(table=1)

res=para_con_2seq(seq1=p1,seq2=p2,seq3=p3,seq4=p4)

o=open(outfile,'w')

h=['id','type']

o.write(sep.join(h)+'\n')

for id in res.keys():

line=[id,res[id]]

print line

line=[str(x) for x in line]

o.write(sep.join(line)+'\n')
