# Supplementary material for: Adaptive Evolution of Energy Metabolism-Related Genes in Hypoxia-Tolerant Mammals
Source: Front Genet. 2017 Dec 7;8:205. doi: 10.3389/fgene.2017.00205 (PMC5725996; doi:10.3389/fgene.2017.00205)
Supplement: Supplementary file 6 [file Image_1.PDF]

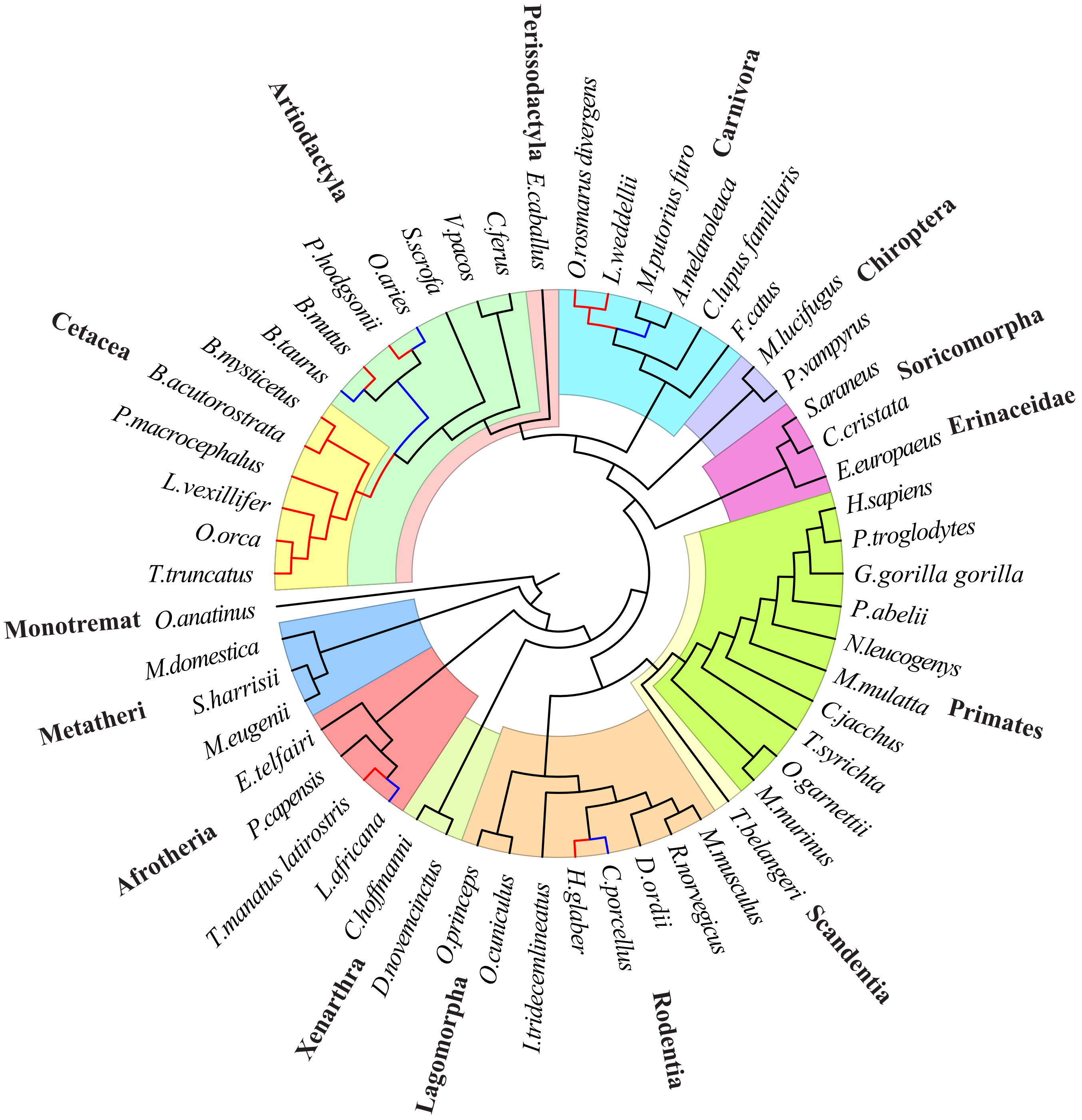

Fig. S1. The tree topology used to conduct the selective pressure analysis in PAML (Murphy et al. 2001). Different orders of mammals were marked with different colors. Red and blue color branches in the tree represented hypoxia tolerant mammals and their sister taxa, respectively. They are used in the branch-site models tests, and results listed in table 1, table S2, and fig. 2.

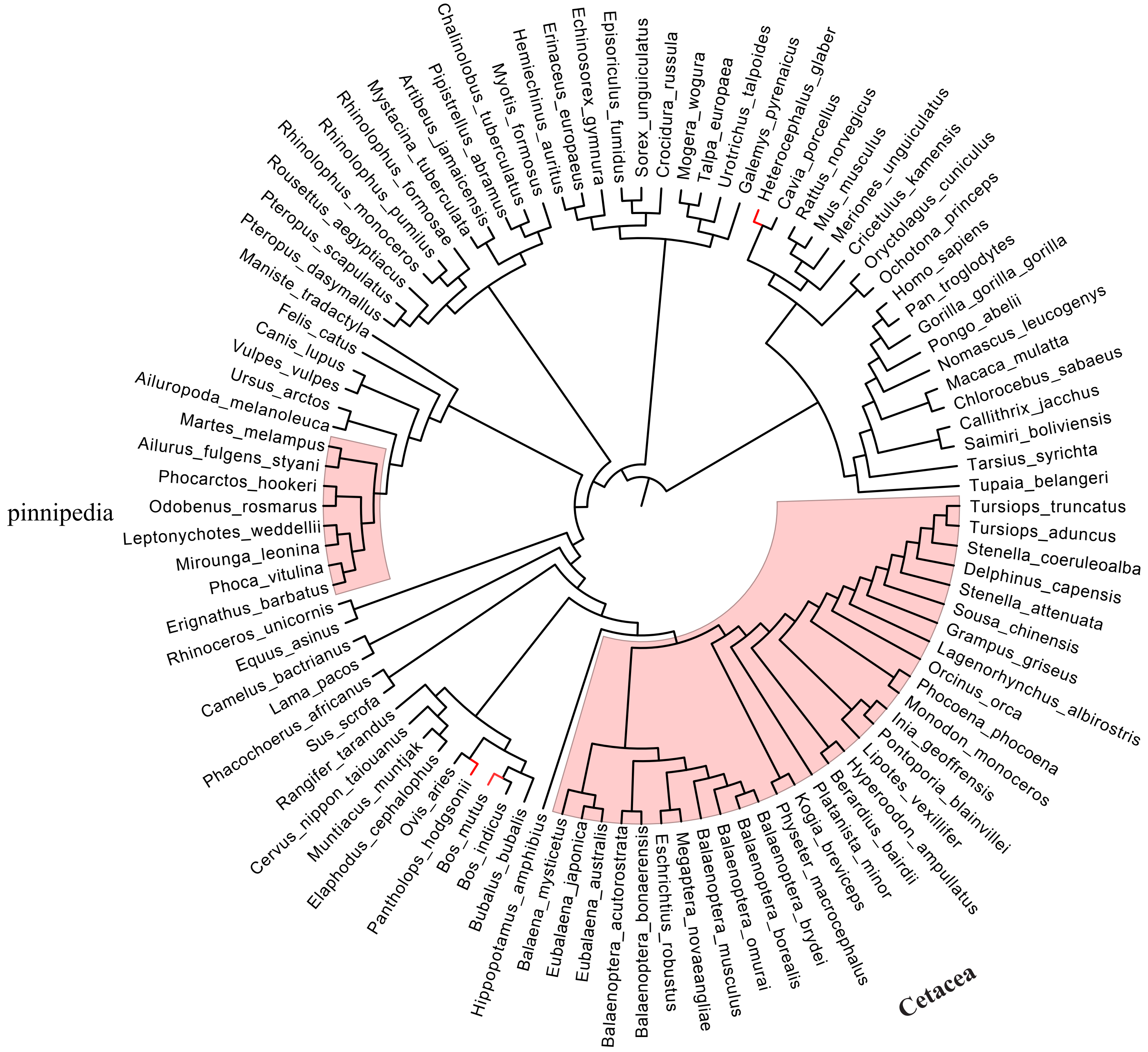

Figure S2. Phylogeny of 101 mammals used for evolutionary analysis of mitochondrial genomes. Hypoxia tolerance species were marked in red.

3.0

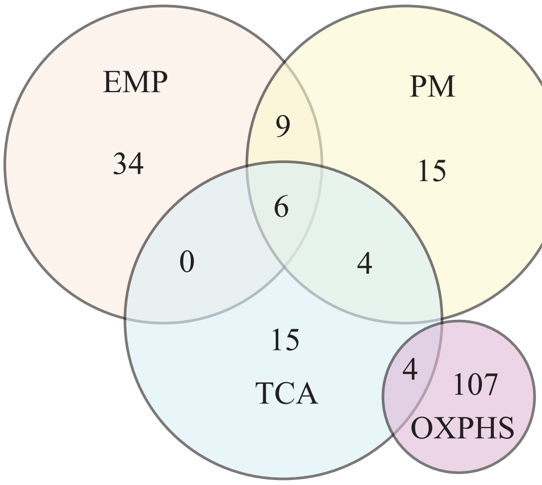

Fig. S3 Gene number are involved in four pathways of Glycolysis / Gluconeogenesis (EMP): ko00010, Citrate cycle pathway (TCA): ko00020, Pyruvate metabolism (PM): ko00620, Oxidative phosphorylation (OXPHS): ko00190 in KEGG database.

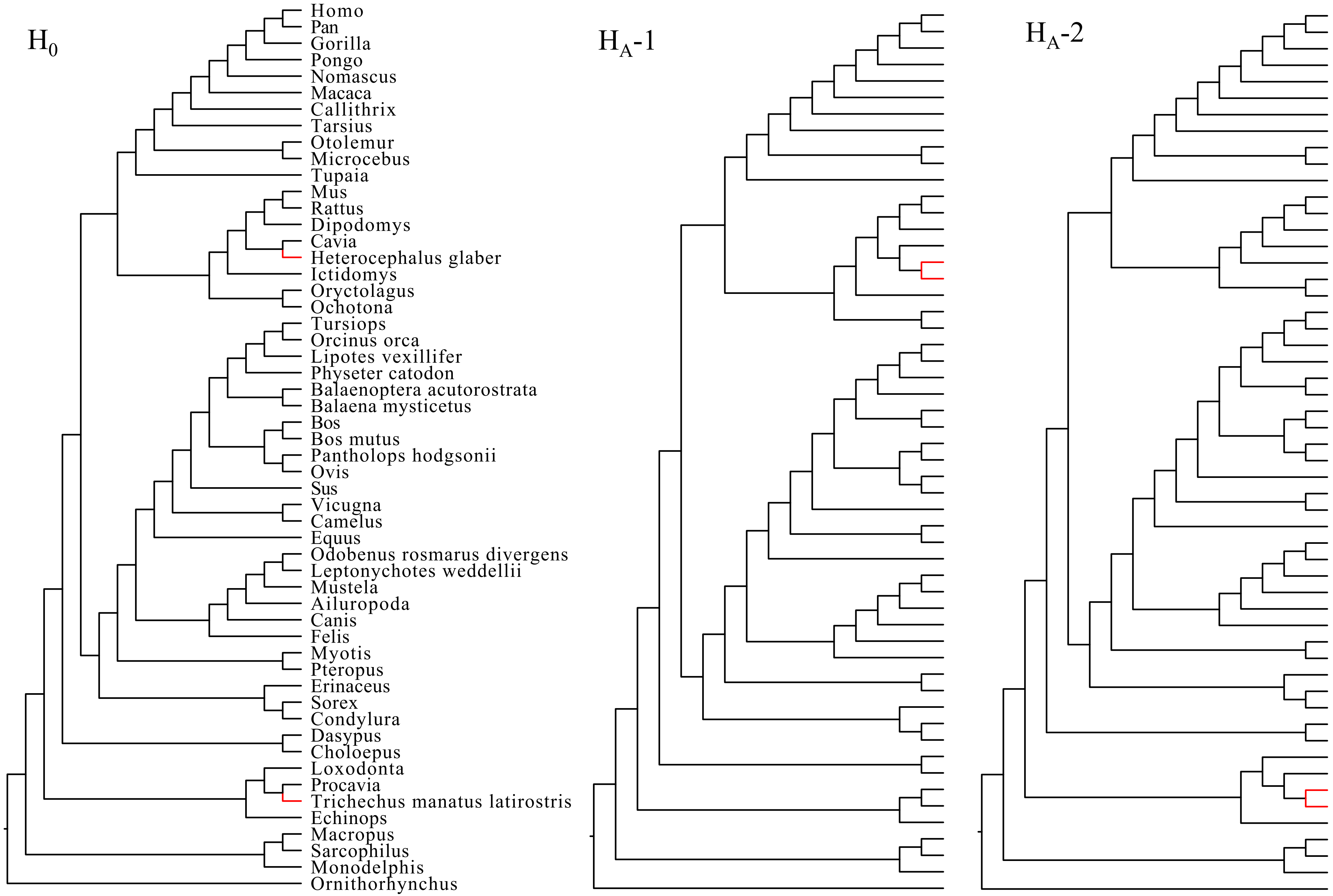

Fig. S4 Null hypothesis (species tree) and other 2 alternative hypotheses examined in the likelihood convergence analyses.

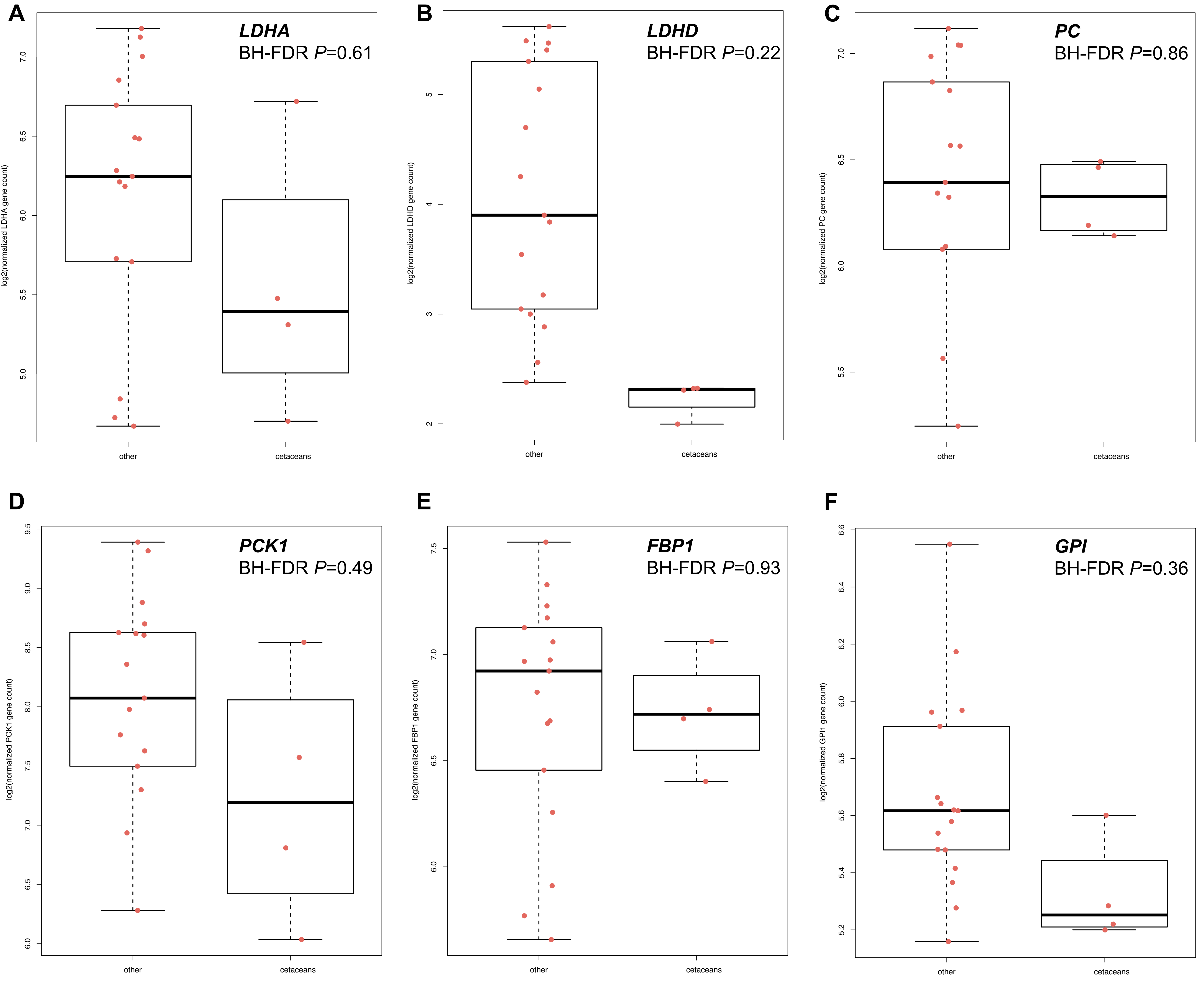

Fig. S5 Liver transcriptomes of energy metabolism genes of cetaceans and other terrestrial mammals

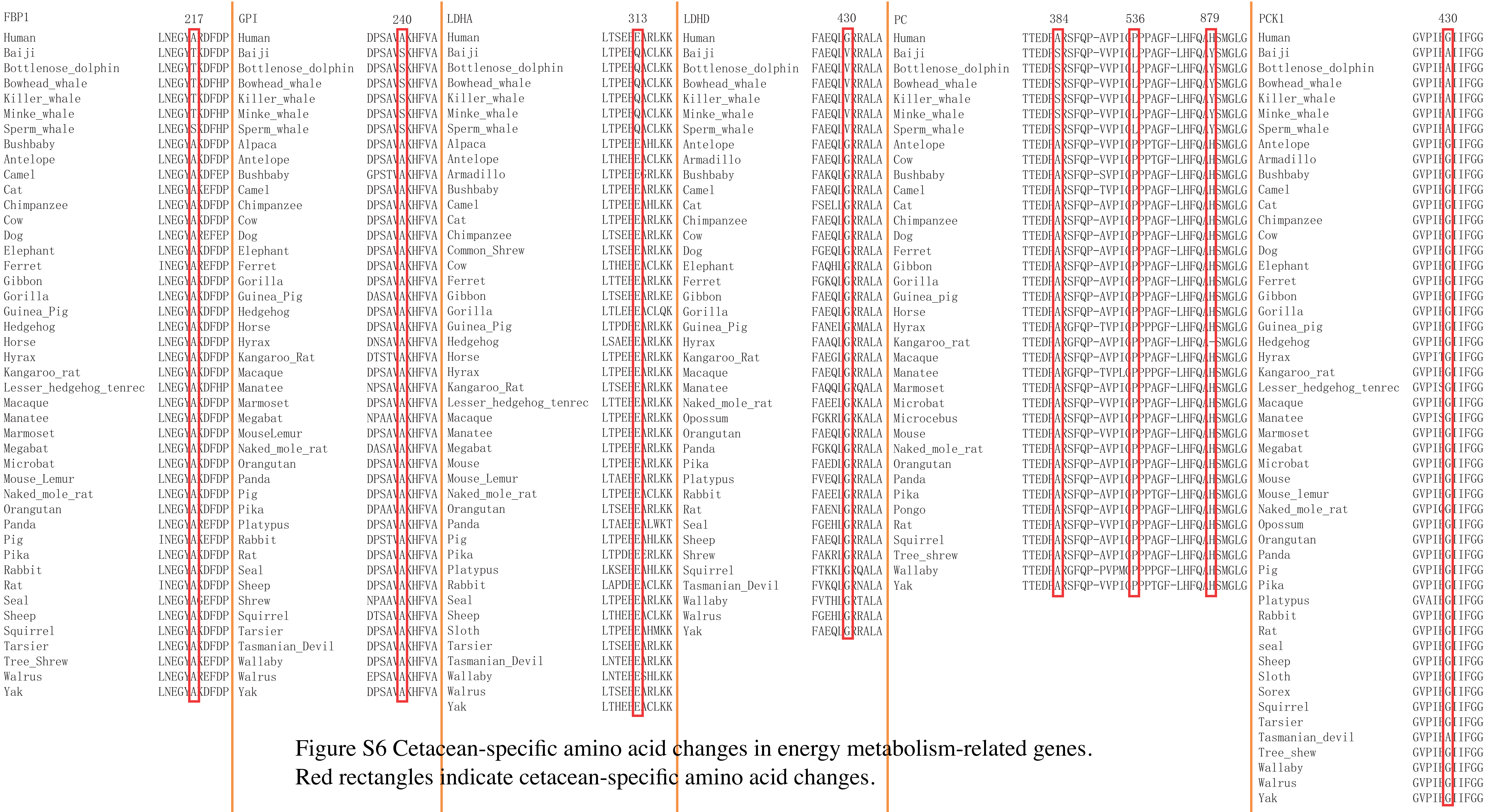

Figure S6 Cetacean-specific amino acid changes in energy metabolism-related genes. Red rectangles indicate cetacean-specific amino acid changes.
